# Sodium-Glucose Co-Transporter-2 Inhibitors in Non-Diabetic Adults With Overweight or Obesity: A Systematic Review and Meta-Analysis

**DOI:** 10.3389/fendo.2021.706914

**Published:** 2021-08-16

**Authors:** Hanrui Zheng, Min Liu, Sheyu Li, Qingyang Shi, Shengzhao Zhang, Yiling Zhou, Na Su

**Affiliations:** ^1^Department of Pharmacy, West China Hospital, Sichuan University, Chengdu, China; ^2^West China School of Pharmacy, Sichuan University, Chengdu, China; ^3^Department of Pharmacy, Mianyang Central Hospital, School of Medicine, University of Electronic Science and Technology of China, Mianyang, China; ^4^Department of Endocrinology and Metabolism, West China Hospital, Sichuan University, Chengdu, China; ^5^Department of Guideline and Rapid Recommendation, Cochrane China Center, MAGIC China Center, Chinese Evidence-Based Medicine Center, West China Hospital, Sichuan University, Chengdu, China; ^6^Department of Pharmacy, Karamay Central Hospital, Karamay, China

**Keywords:** SGLT2 inhibitors, obesity, overweight, non-diabetic adults, meta-analysis

## Abstract

**Background:**

Sodium-glucose-cotransporter-2 (SGLT2) inhibitors have proven to be effective in improving glycemic control and lowering body weight in patients with type 2 diabetes mellitus. However, the efficacy and safety on weight loss in adults with overweight or obesity but not diabetes remain unclear. In this article, we aimed to identify the efficacy and safety of SGLT2 inhibitors in adults with overweight or obesity but not diabetes in randomized controlled studies (RCTs).

**Methods:**

We searched for RCTs concerning SGLT2 inhibitors in adults with overweight or obesity but not diabetes in Medline (Ovid SP), Embase (Ovid SP), Cochrane Central Register of Controlled Trials (Ovid SP), and ClinicalTrials.gov up to February 2021. The primary outcomes were changes in body weight and body mass index (BMI). Trial sequential analysis (TSA) was used to test the reliability of the primary outcomes. We analyzed the data using Review Manager 5.3 and pooled data to calculate the mean differences (MDs) or the relative risk (RR). We assessed the evidence quality of evidence of outcomes according to GRADE.

**Results:**

Six randomized controlled trials involving 872 individuals were included in the meta-analysis. Compared to the placebo group, the SGLT2 inhibitors group had statistically significant reductions in absolute changes in body weight (MD: -1.42 kg, 95% CI: -1.70 to -1.14; P<0.00001) and BMI (MD: -0.47 kg/m2, 95% CI: -0.63 to -0.31; P<0.00001) in SGLT2 inhibitors group, as indicated by TSA. However, no significant benefits were observed in the SGLT2 inhibitors group in terms of waist circumference (MD: -1.34 cm, 95%CI: -2.75 to 0.07; Z=1.86, P=0.06) compared with the placebo group. The GRADE profiles indicated very low-quality evidence for body weight change and low-quality evidence for BMI change. SGLT2 inhibitors were generally safe and well tolerated.

**Conclusion:**

SGLT2 inhibitors could be used in selected adults with overweight and obesity but not diabetes if they are at low risk of genital infection and urinary infection. Further studies are warranted to confirm the efficacy and safety of SGLT2 inhibitors in adults with overweight or obesity but not diabetes for long-term weight management.

**Systematic Review Registration:**

[https://www.crd.york.ac.uk/prospero/#loginpage], identifier [PROSPERO, CRD42021252931]

## Introduction

Overweight and obesity are major risk factors for several diseases, such as hypertension, dyslipidaemia, type 2 diabetes, cardiovascular disease, osteoarthritis, obstructive sleep apnoea, fatty liver disease cancers and other diseases (1, 2). Moderate weight loss (5% of body weight) can improve glycaemic control and insulin homeostasis and mitigate cardiovascular risk factors associated with overweight and obesity (3). In 2016, the World Health Organization reported that more than 1.9 billion adults were affected by overweight, of whom 650 million adults were affected by obesity (4), and that over 2.8 million deaths were attributable to overweight or obesity per year. The issues once considered specific to developed countries are now also on the rise in developing countries, especially in urban settings, which require additional healthcare interventions (5).

The management of overweight and obesity is challenging but imperative. Clinical practice guidelines have recommended lifestyle interventions such as diet, exercise, and behavioural modification for weight management. Bariatric surgery and/or pharmacological treatment have also been recommended based on lifestyle interventions. Although bariatric surgery is an effective treatment option, it is invasive, relatively expensive, available only to a limited population, and may be associated with adverse consequences. Weight loss medications for obesity include phentermine, topiramate/phentermine, lorcaserin, orlistat, naltrexone/bupropion and liraglutide, often with some side effects for long-term use (6).

Sodium-glucose transporter 2 (SGLT2) inhibitors are a novel class of oral therapeutic medications that have been approved for the treatment of type 2 diabetes mellitus by the Food and Drug Administration (FDA) (7). SGLT2 is mostly expressed in the renal proximal convoluted tubule. Its inhibition leads to a decreased renal threshold for glucose excretion (RTG) and increased urinary glucose excretion (UGE), resulting in mild diuresis and a net caloric loss. SGLT2 inhibitors have been shown to be successful in improving glycaemic control and lowering body weight (8). A great deal of evidence has indicated that SGLT-2 inhibitors have strong effects on body weight in patients with diabetes mellitus and can be used as potential agents for obesity management (9, 10). However, the efficacy and safety of SGLT2 inhibitors therapy in adults with overweight or obesity but not diabetes remain unknown.

Therefore, we conducted a systematic review and meta-analysis of SGLT2 inhibitors in randomized controlled trials (RCTs) to assess whether SGLT2 inhibitors could lead to weight loss in adults with overweight or obesity but not diabetes.

## Materials and Methods

This systematic review and meta-analysis was written in accord with the Preferred Reporting Items for Systematic Reviews and Meta Analyses (PRISMA) (11). This systematic review was registered on International Prospective Register of Systematic Review (PROSPERO, CRD42021252931).

### Literature Search

An extensive search for RCTs in Medline (Ovid SP), Embase (Ovid SP), Cochrane Central Register of Controlled Trials (Ovid SP), for studies published from the creation time of databases until February 20^th^, 2021, using the keywords: “Sodium-Glucose Transporter 2”, “sodium glucose cotransporter 2 inhibitors”, “canagliflozin”, “dapagliflozin”, “empagliflozin”, “ipragliflozin”, “tofogliflozin”, “luseogliflozin”, “sergliflozin”, “remogliflozin”, “ertugliflozin”, “sotagliflozin”, “overweight”, “obesity” and “obese” (**Supplementary Information 2**). ClinicalTrial.gov was screened for potentially eligible studies. The reference lists of relevant published researches investigating the use of SGLT2 inhibitors in non-diabetes with overweight or obesity were also reviewed for potentially relevant studies. We contacted authors by email if the full-text was not available or if the outcomes were not enough.

### Study Selection

We included studies meeting the following criteria: (1) Participants: adults with overweight or obesity but not diabetes undergoing SGLT2 inhibitors based on the study definition; (2) Interventions/comparisons: using SGLT2 inhibitors as a monotherapy and placebo as the control. All included participants received standardized advice on diet and physical activity throughout the trial; (3) Outcomes: reporting one of the primary outcomes of interest, namely body weight and body mass index (BMI). Weight loss ≥ 5%, Waist circumference (WC), Hip circumference (HC), Waist/hip ratio (W/H) and adverse events were secondary outcomes. The adverse events included general adverse events and serious adverse events; (4) Study design: randomized controlled trials (RCTs) limited to the English language without restrictions of study size, follow-up length or publication year. No ethical approval and no contact with individual patients were required. The exclusion criteria were as follows: (1) including participants with pregnant; (2) animal experiments; (3) studies published in a language other than Chinese or English; (4) published as abstract only; (5) including patients with prediabetes.

### Data Extraction

All retrieved literatures were identified by two independent reviewers (HZ and ML) and data were extracted by a pre-defined form. Any discrepancies were resolved by discussion with a 3rd reviewer (NS) when necessary. We extracted the following data: (1) the last name of the first author, publication year; (2) sample size, follow-up length, intervention and comparison; (3) the characteristics of participants’ age, gender, country; body weight; BMI; (4) outcomes and (5) funding. SGLT2 inhibitors with diverse doses were separated to several trials. If a study contained more than one SGLT-2 inhibitors or more than one dose of SGLT-2 inhibitors, we separated it to different trials, one of which only included one of SGLT-2 inhibitors group with only one dose.

### Quality Assessment

Two independent reviewers (HZ and ML) assessed the risk of bias of the included studies according to the Cochrane Handbook for Systematic Reviews of Interventions (version 5.1), and the disagreement were resolved by consulting the 3rd reviewer (NS). We assessed the quality of the included studies concerning 7 aspects including random sequence generation, allocation concealment, blinding of participants and personnel, blinding of outcome assessment, incomplete outcome data, and selective reporting and other bias (12). Each of them was judged as low, high or unclear risk. Grading of Recommendations Assessment, Development and Evaluation (GRADE) (13, 14) tool was used to assess the evidence quality and provide evidence for future guidelines, concerning inconsistency, indirectness, imprecision, and other bias.

### Statistical Analyses

All data were analyzed by Revman software (version 5.3; Cochrane Collaboration). Trial sequential analysis (TSA) (version 0.9.5.10 Beta; Copenhagen Trial Unit, Copenhagen, Denmark) was used for assessing the risk of type I and II errors, quantifying the statistical reliability of data in the meta-analysis and control this potential risk. TSA was conducted for primary outcome. An overall 5% type I error was maintained with a power of 80%. All dichotomous data were calculated as a relative risks (RR) with accompanying 95% confidence interval (CI), while all continuous data were calculated as a mean difference (MD) with accompanying 95% CI. Chi-squared test and I^2^ statistic was used to assess the degree of statistical heterogeneity. When statistical heterogeneity occurred (P value <0.10, I^2^ >25%), a random-effect model was used and possible sources of heterogeneity were explored, otherwise, a fixed-effects model was used. Subgroup analyses according to the drugs of SGLT2 inhibitors were pursued. Publication bias was assessed using funnel plots and Egger’ s test (*meta* package in R v4.1.0). A sensitivity analysis was conducted removing a single study at a time in an iterated manner and using different pooling methods.

## Results

### Study Search and Trial Characteristics

**Figure 1:**
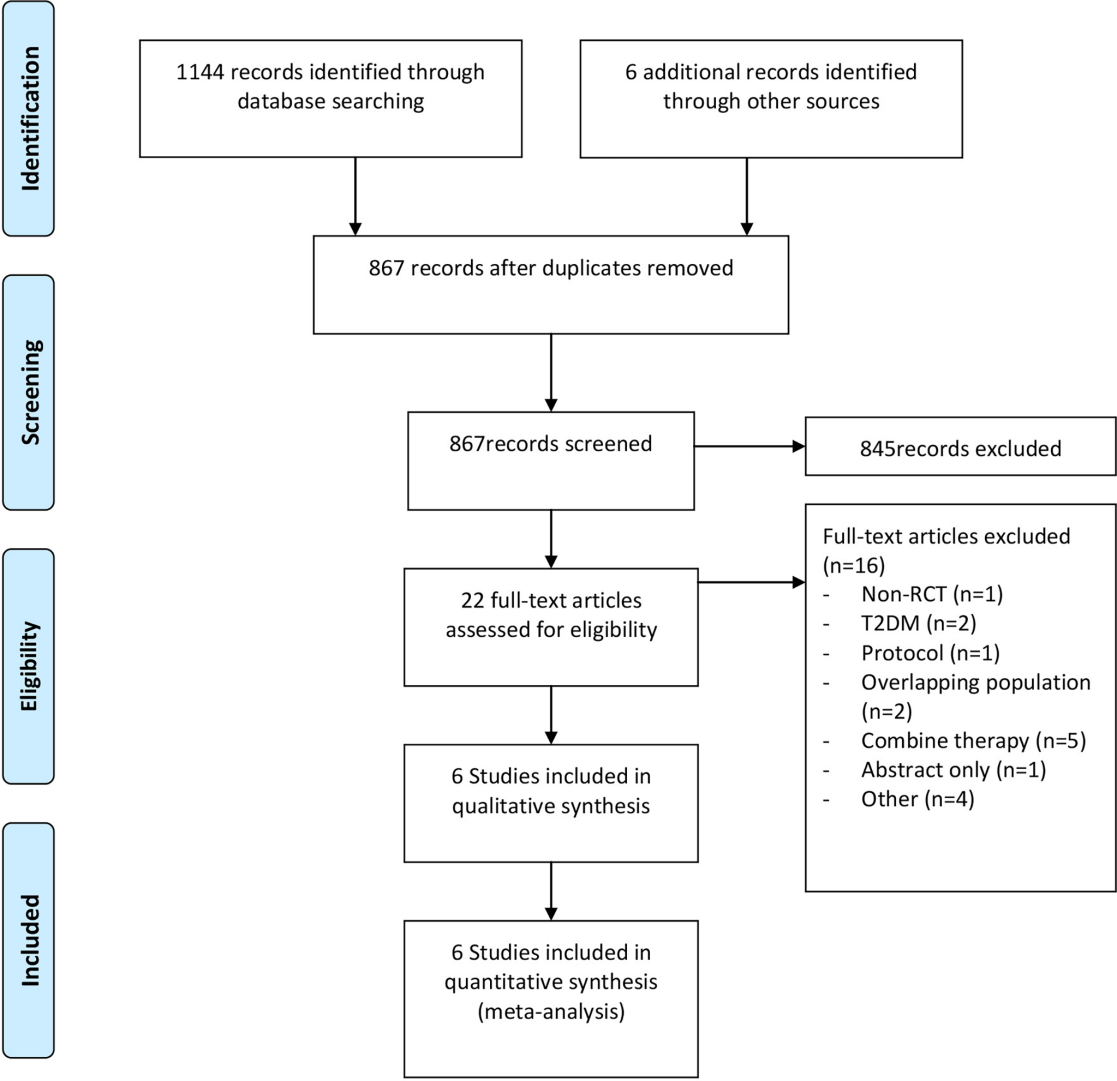
Flow diagram for study identification and inclusion.

As illustrated in **Figure 1**, a total of 1150 studies were identified, among which 6 were from the ClinicalTrial.gov. Owing to repetition, 283 studies were omitted. After screening the titles and abstracts, 845 studies were excluded, and 22 potentially eligible studies were reviewed by full-text. Full-text reviewed excluded 16 studies (**Supplementary Information 2**). Eventually, six studies involving 872 participants (15–20) were included in the final meta-analysis and all of the included studies were reported in English.

The characteristics of the included studies are reported in **Table 1**. All six studies (15–20) focused on adults with overweight or obesity but not diabetes. Participants in these studies came from four regions, including the United States, Puerto Rico, the United Kingdom, Denmark. Five studies (16–18, 20) were randomized, placebo-controlled studies, and one (19) was a pilot trial. Of these, 2 RCTs (16, 18) (n=720) evaluated canagliflozin (50 to 300 mg once daily), 2 RCTs (15, 17) (n=86) evaluated dapagliflozin (10 mg once daily), 2 RCTs (19, 20) (n=45) evaluated sergliflozin (500 to 1000 mg three times daily), and 1 RCT (19) (n=21) evaluated remogliflozin (250 mg three times daily), while all control groups were placebo. The follow-up periods ranged from 2 to 26 weeks. The mean ages of the included individuals ranged from 18.0 to 61.4 years old. The proportion of men was reported to be 21.25% (143 to 673). The mean body weight varied from 68.0 to 105.0 kg.

**Table 1:** Baseline characteristics of each included study (n=6).

Author (year)	I	C	Number		Male (%)		Mean age (years)		Mean body weight (kg)		Mean BMI (kg/m²)		Patients	Country	Follow-up(weeks)	Outcomes	Funding
			T	C	T	C	T	C	T	C	T	C					
Færch2021 (15)	DAPA 10mg qd	PBO	30	30	43	40	61.4 ± 8.5	57.2 ± 9.9	M:103.7 ± 17.6	M:99.0 ± 15.1	M:31.6 ± 3.8	M:30.4 ± 3.2	Overweight/Obese with PreDM	Denmark	13	BW,BMI,WC,W/H	Novo Nordisk Foundation,AstraZeneca AB
									F:82.5 ± 13.7	F:92.1 ± 25.3	F:30.4 ± 5.1	F:34.0 ± 8.4					
Hollander2017 (16)	CANA 300mg qd	PBO	84	82	19	18.3	45.2 ± 11.0	44.8 ± 11.1	103.3 ± 19.1	104.3 ± 18.2	37.3 ± 4.7	38.0 ± 5.2	Overweight/Obese Without DM	US	26	BW,BW(loss) ≥5%,BMI,WC	Janssen Research & Development, LLC
Gonzalez-Ortiz2017 (17)	DAPA 10mg qd	PBO	13	13	15.4	15.4	46.5 ± 5.2	45.0 ± 6.8	68.0 ± 4.6	73.0 ± 8.2	27.3 ± 2.0	27.3 ± 1.6	Overweight Without DM	NR	12	BW,BMI,WC	
Bays2014 (18)	CANA 50mg qd	PBO	98	89	12	16	44.9 ± 11.8	45.1 ± 11.9	98.1 ± 16.0	102.2 ± 19.9	36.6 ± 5.3	36.6 ± 5.5	Overweight/Obese Without DM	US and Puerto Rico	12	BW,BW(loss) ≥5%,BMI, WC,HC,W/H	Janssen Global Services, LLC
	CANA 100mg qd	PBO	93	89	12	16	45.8 ± 11.0	45.1 ± 11.9	105.0 ± 16.6	102.2 ± 19.9	37.9 ± 5.1	36.6 ± 5.5					
	CANA 300mg qd	PBO	96	89	10	16	43.5 ± 11.0	45.1 ± 11.9	100.2 ± 18.0	102.2 ± 19.9	36.9 ± 5.3	36.6 ± 5.5					
Napolitano2014 (19)	REMO 250mg tid	PBO	9	12	73		42 ± 13.0		101 ± 14.6		33 ± 2.4		Healthy obese	UK	8	BW,BMI,WC,HC	Glaxo Smith Kline
	SER 1000mg tid	PBO	9	12													
Hussey2010 (20)	SER 500mg tid	PBO	6	6	44.4		18-55		NR		25-35		Healthy Overweight/Obese	US	2	BW	Glaxo Smith Kline
	SER 1000mg tid	PBO	6	6													

DM, diabetes mellitus; NA, not applicable; I, Intervention C, Control; DAPA, dapagliflozin; CANA, canagliflozin; REMO, remogliflozi; SER, sergliflozin; PBO, placebo; BMI, body mass index; BW, body weight; WC, Waist circumference; HC, Hip circumference; W/H, Waist/hip ratio.

### Quality of Bias Control

Two studies (15, 16) were assessed low risk for random sequence generation bias, while four (17–20) were unclear. Four studies (16–18, 20) were assessed low risk bias for blinding of participants and personnel, while two (15, 19) was high risk bias. The assessment results of quality were shown in **Supplementary Figures 1**, **2**.

**Figure 2:**
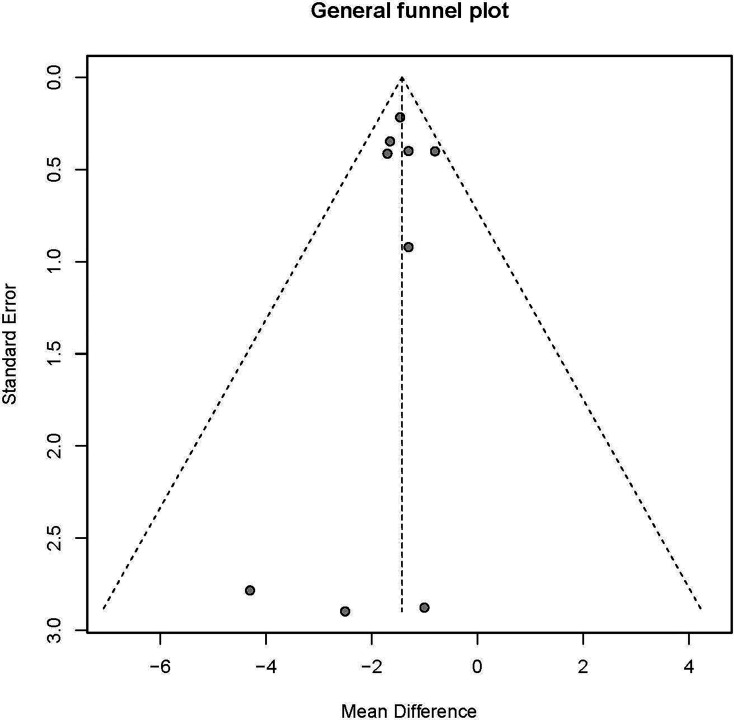
General funnel plot analysis.

No publication bias was found from Egger’s test (t = −0.08, P = 0.939), and the funnel plot showed a symmetric distribution (**Figure 2**).

### Meta-Analyses of Body Weight Change

**Figure 3:**
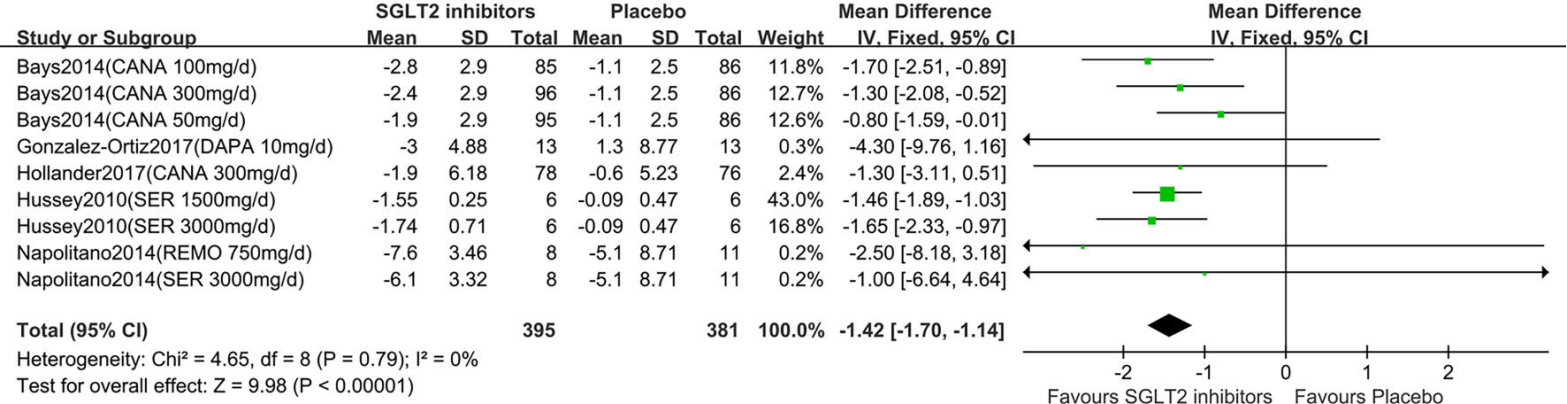
Changes of body weight in patients receiving SGLT2 inhibitors *versus* placebo. CI, confidence interval; IV, inverse variance; SD, standardized deviation.

Six studies displayed body weight change (15–20). One study reported that participants in the dapagliflozin group had reduced body weight by −1.1 kg (95% CI: −2.6 to 0.3) compared with the control (15). Others (16–20) were summarized for meta-analysis. Compared to placebo, SGLT2 inhibitors were associated with a statistically significant reduction in body weight (MD: 1.42 kg, 95% CI: -1.70 to -1.14; Z=9.98, P<0.00001), which was homogeneous (I^2^ = 0%, P = 0.80) (**Figure 3**). This was very low-quality evidence that was downgraded one level for risk of bias, one level for indirectness and one level for imprecision (**Table 2**).

**Table 2:** The GRADE profiles: SGLT2 inhibitors compared to placebo in overweight or obese individuals without diabetes.

Outcomes	Illustrative comparative risks (95% CI)		Relative effect (95% CI)	No. of Participants (studies)	Quality assessment						Quality of the evidence (GRADE)
	Assumed risk	Corresponding risk			Design	Risk of bias	Inconsistency	Indirectness	Imprecision	Other considerations	
	Placebo	SGLT2									
**Body weight**		The mean BW in the intervention groups was		776	RCT	serious^1^	no serious inconsistency	serious^2^	serious^3^	none	very low quality^1,2,3^
		**1.42 lower**		(5 studies)							
		(1.7 to 1.14 lower)									
**BMI**		The mean BMI in the intervention groups was		749	RCT	serious^1^	no serious inconsistency	serious^2^	no serious imprecision	none	low quality^1,2^
		**0.47 lower**		(4 studies)							
		(0.63 to 0.31 lower)									
**Weight loss ≥5%**	**Study population**		**RR 1.68**	644	RCT	no serious risk of bias	no serious inconsistency	serious^4^	no serious imprecision	none	moderate quality evidence^4^
			(1.11 to 2.54)	(2 studies)							
	**98 per 1000**	**165 per 1000**									
		(109 to 250)									
	**Moderate**										
	**81 per 1000**	**136 per 1000**									
		(90 to 206)									
**WC**		The mean WC in the intervention groups was		540	RCT	serious^5^	no serious inconsistency	serious^5^	no serious imprecision	none	low quality^5^
		**1.34 lower**		(3 studies)							
		(2.75 lower to 0.07 higher)									
**HC**		The mean HC in the intervention groups was		508	RCT	serious^1^	no serious inconsis tency	serious^2^	no serious impreci sion	none	low quality^1,2^
		**1.86 lower**		(2 studies)							
		(3.33 to 0.38 lower)									
**W/H**		The mean W/H in the intervention groups was		470	RCT	no serious risk of bias	no serious inconsistency	serious^4^	no serious imprecision	none	moderate quality^4^
		**0.01 higher**		(3 studies)							
		(0 to 0.02 higher)									

CI, Confidence interval; RR, Risk ratio; BMI, Body mass index; Weight loss ≥5%, Proportion of subjects who lost 5% of baseline body weight at Week 12; WC, Waist circumference; HC, Hip circumference; W/H, Waist/hip ratio; GRADE, Grading of Recommendations Assessment, Development and Evaluation. High quality: further research is very unlikely to change our confidence in the estimate of effect. Moderate quality: further research is likely to have an important impact on our confidence in the estimate of effect and may change the estimate. Low quality: further research is very likely to have an important impact on our confidence in the estimate of effect and is likely to change the estimate. Very low quality: we are very uncertain about the estimate.

^1^Downgraded one level for risk of bias [Napolitano2014 (19): high risk of bias for blinding].

^2^Downgraded one level for risk of bias [Bays 2014 (18); Napolitano 2014 (19)].

^3^Very small samples sizes in Hussey 2010 (20).

^4^Downgraded one level for risk of bias [Bays 2014 (18)].

^5^No explanation was provided.

In the subgroup analyses of SGLT2 inhibitors, we found that the individuals in the canagliflozin and sergliflozin groups had a statistically significant reduction in body weight compared to those in the placebo group (MD: -1.26 kg, 95% CI: -1.70 to -0.82; MD: -1.51 kg, 95% CI: -1.87 to -1.15, respectively), while the individuals in the dapagliflozin and remogliflozin groups had no statistically significant reduction in body weight compared to those in the placebo group (MD: -4.30 kg, 95% CI: -9.76 to 1.16; MD: -2.50 kg, 95% CI: -8.18 to 3.18, respectively). TSA showed that the pooled results (Z curve) crossed the conventional boundary of benefit and reach the required information size (RIS=316). It confirmed that the SGLT2 inhibitors could significantly lowered body weight (**Supplementary Figure 3**).

### Meta-Analyses of BMI Change

**Figure 4:**
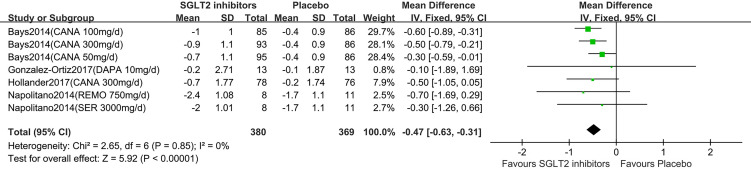
Changes of BMI in patients receiving SGLT2 inhibitors *versus* placebo. BMI, body mass index; CI, confidence interval; IV, inverse variance; SD, standardized deviation.

Five studies displayed BMI change (15–19). One study reported that participants in the dapagliflozin group had reduced BMI by −0.3 kg/m^2^ (95%CI: −0.8 to 0.1) compared with the control (15). Others (16–19) were summarized for meta-analysis. Compared to placebo, SGLT2 inhibitors were associated with a statistically significant reduction in BMI (MD: -0.47 kg/m^2^, 95%CI: -0.63 to -0.31;Z=5.92, P<0.00001), which was homogeneous (I^2^ = 0%, P = 0.85) (**Figure 4**). This was low quality evidence that was downgraded one level for risk of bias and one level for indirectness (**Table 2**).

In the subgroup analyses of SGLT2 inhibitors, we found that the individuals in the canagliflozin groups had a statistically significant reduction in BMI compared to those in the placebo group (MD: -0.47 kg/m^2^, 95%CI: -0.63 to -0.31), while the individuals in the dapagliflozin, remogliflozin and sergliflozin groups had no statistically significant reduction in BMI (MD: -0.10 kg/m^2^, 95% CI: -1.89 to 1.69; MD: -0.70 kg/m^2^, 95% CI: -1.69 to 0.29; MD: -0.30 kg/m2; 95% CI: -1.26 to 0.66, respectively).

TSA showed that the pooled results (Z curve) crossed the conventional boundary of benefit and reach the required information size (RIS=218). It confirmed that the SGLT2 inhibitors could significantly lowered BMI (**Supplementary Figure 4**).

### Meta-Analyses of Weight Loss ≥5%

**Figure 5:**
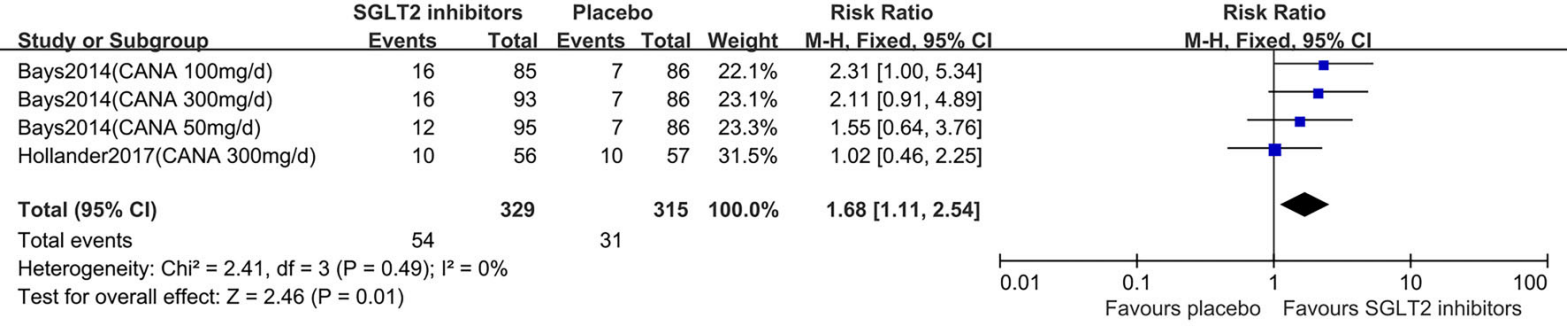
Changes of weight loss ≥5% in patients receiving SGLT2 inhibitors *versus* placebo. CI, confidence interval; M-H, Mantel-Haenszel.

Two studies (16, 18) were summarized for meta-analysis. Compared to placebo, SGLT2 inhibitors were associated with a statistically significant greater in the proportion of individuals achieved weight loss over 5% (RR: 1.68, 95% CI: 1.11 to 2.54; Z=2.46, P=0.01), and it was homogeneous (I^2^ = 0%, P = 0.49) (**Figure 5**). This was moderate quality evidence that was downgraded one level for indirectness (**Table 2**).

### Meta-Analyses of Waist Circumference Change

**Figure 6:**
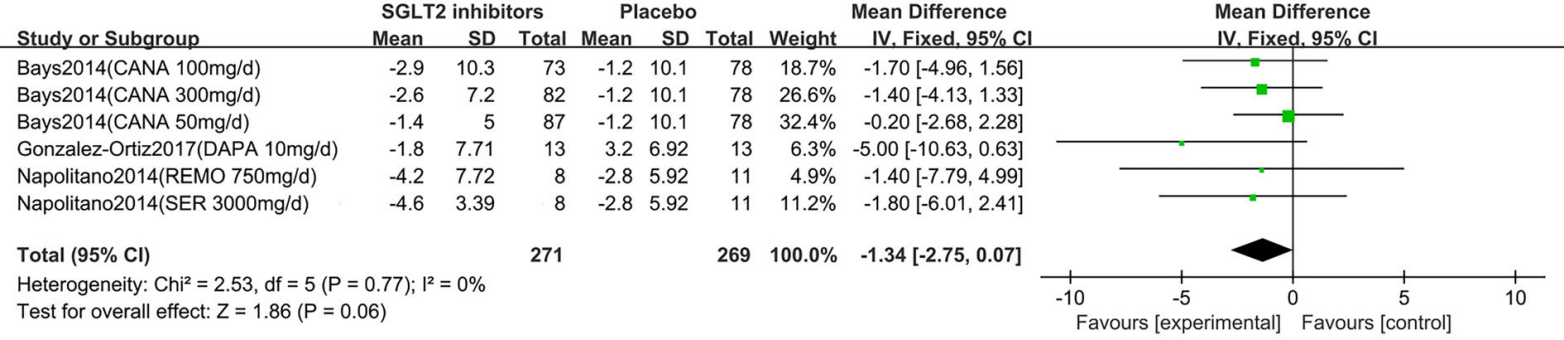
Changes of waist circumference in patients receiving SGLT2 inhibitors *versus* placebo. CI, confidence interval; IV, inverse variance; SD, standardized deviation.

Five studies displayed waist circumference change (15–19). One study reported that participants in the dapagliflozin group had reduced waist circumference by −2.4 cm (95% CI: −4.8 to 0.0) compared with control (15). The others (16–19) were summarized for meta-analysis. Compared to placebo, SGLT2 inhibitors were not associated with a statistically significant reduction in waist circumference (MD: -1.34 cm, 95% CI: -2.75 to 0.07; Z=1.86, P=0.06), which was homogeneous (I^2^ = 0%, P = 0.77) (**Figure 6**). This was low quality evidence that was downgraded one level for risk of bias and one level for indirectness (**Table 2**).

In the subgroup analyses of SGLT2 inhibitors, we found that the individuals in the canagliflozin, dapagliflozin, remogliflozin and sergliflozin groups had no statistically significant reduction in waist circumference compared to those in the placebo group (MD: -0.97 cm, 95% CI: -2.57 to 0.63; MD: -5.00 cm, 95% CI: -10.63 to 0.63; MD: -1.40 cm, 95% CI: -7.79 to 4.99; MD: -1.80 cm, 95% CI: -6.01 to 2.41, respectively).

### Meta-Analyses of Hip Circumference Change

**Figure 7:**
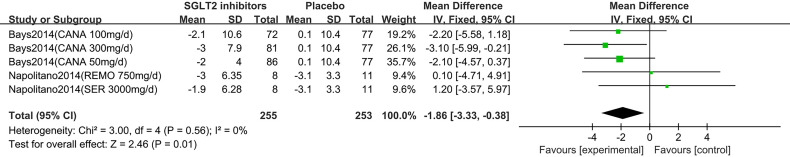
Changes of hip circumference in patients receiving SGLT2 inhibitors *versus* placebo. CI, confidence interval; IV, inverse variance; SD, standardized deviation.

Two (18, 19) studies were summarized for meta-analysis. As shown in **Figure 7**, compared to placebo, SGLT2 inhibitors were associated with a statistically significant reduction in hip circumference (MD: -1.86 cm, 95% CI: -3.33 to -0.38; Z=2.46, P=0.01), which was homogeneous (I^2^ = 0%, P = 0.56). This was low quality evidence that was downgraded one level for risk of bias and one level for indirectness (**Table 2**).

In the subgroup analyses of SGLT2 inhibitors, we found that individuals in the canagliflozin group had a statistically significant reduction in hip circumference compared to those in the placebo group (MD: -2.45 cm, 95% CI: -4.09 to -0.80), while the individuals in the remogliflozin and sergliflozin groups had no statistically significant reduction in hip circumference (MD: 0.10 cm, 95% CI: -4.71 to 4.91; MD: 1.20 cm, 95% CI: -3.57 to 5.97, respectively).

### Meta-Analyses of Waist/Hip Ratio Change

**Figure 8:**
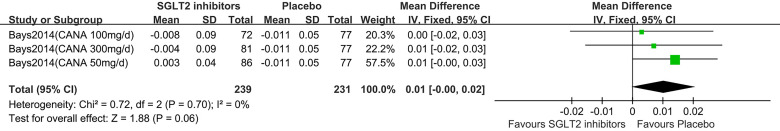
Changes of waist hip ratio in patients receiving SGLT2 inhibitors *versus* placebo. CI, confidence interval; IV, inverse variance; SD, standardized deviation.

Two studies displayed waist/hip ratio change (15, 18). One study reported that participants in dapagliflozin group had on average reduced waist/hip ratio (MD: −0.02, 95% CI −0.04 to 0.01) compared with control (15). One study (18) was summarized for meta-analysis. It is no significant difference of waist/hip ratio was observed between SGLT2 inhibitors and control group (MD: 0.01, 95% CI: -0.00 to 0.02; Z=1.88, P=0.06), which was homogeneous (I^2^ = 0%, P = 0.70) (**Figure 8**). This was moderate quality evidence that was downgraded one level for indirectness (**Table 2**).

### Adverse Events

SGLT2 inhibitors were generally well tolerated. Few serious adverse events were observed and none were considered related to study drug. It was shown that SGLT2 inhibitors increased the number of participants who withdrew or dropped out from studies (**Table 3**). Hypoglycemia, urinary tract infection, or sinusitis observed with SGLT2 inhibitors were similar to those reported in the placebo group. However, individuals assigned to SGLT2 inhibitors treatment suffered more genital mycotic infection, vulvovaginal mycotic infection and nausea than those in the placebo group. No adverse events of fractures, diabetic ketoacidosis (DKA) and cardiovascular safety were reported in the studies included here.

**Table 3:** Adverse events reported in all included studies.

Adverse events	Numbers (Studies)	SGLT2 inhibitors		Control		Relative risk (95% CI)
		Events	Total	Events	Total	
AEs leading to discontinuation	4	22	401	9	385	2.25 (1.08 to 4.68)
hypoglycemia	1	3	287	6	267	0.49 (0.13 to 1.83)
Urinary tract infection	5	31	420	20	398	1.45 (0.83 to 2.54)
Genital mycotic infection	4	51	393	9	374	5.36 (2.72 to 10.59)
Vulvovaginal mycotic infection	2	31	371	3	349	9.14 (3.00 to 27.78)
Nausea	2	23	371	6	349	3.54 (1.47 to 8.53)
Sinusitis	2	11	371	3	349	2.67 (0.90 to 7.95)

CI, confidence interval.

## Discussion

SGLT2 inhibitors, including canagliflozin, dapagliflozin, empagliflozin, and so on, have proven efficacy when used to treat type 2 diabetes, and all of them were considered effective in reducing body weight (10). Weight loss not only can reduce the risk of cardiovascular disease and endocrine metabolism disease, but also improved the fertility. A prospective cohort study in Boston indicated short term weight loss (3kg) was related to higher MII oocytes yield in women with obesity or overweight undergoing assisted reproductive technology (21). This meta-analysis involving 872 individuals showed that SGLT2 inhibitors may reduce body weight in adults with overweight or obesity but not diabetes, and the result is similar to that of a previous meta-analysis, which showed a reduction in body weight (MD: 1.74 kg, 95% CL: -2.03 to -1.45) compared with placebo in diabetes. Additionally, SGLT2 inhibitors also seemed helpful in reducing BMI but had no beneficial effects on waist circumference control compared with placebo. The subgroup analyses suggested that the weight reduction effect of canagliflozin and sergliflozin may not act in a dose-response manner.

SGLT2 inhibitors were generally well tolerated in previous studies in the type 2 diabetes population (9, 10). The major adverse reactions were genital mycotic infection and urinary infection (16, 18), and they were considered mild to moderate in severity. Compared to placebo, SGLT2 inhibitors were associated with meaningful differences in the incidences of genital mycotic infection and nausea, and particular attention was given to the higher rates of vulvovaginal mycotic infection in women in the SGLT2 inhibitors group, which may be attributable to increased UGE resulting in an increase in vulvovaginal Candida growth (22). No treatment-related fractures or DKA were reported in any group. Reporting of cardiovascular safety was also absent in the included studies, and SGLT2 inhibitors were recently proven to reduce the risk of heart failure in patients with type 2 diabetes by the EMPA-REG OUTCOME study and CANAS study (23, 24).

The exact mechanisms by which SGLT2 inhibitors reduce body weight are not completely understood. Recent clinical studies indicated that the weight loss effect observed with SGLT2 inhibitors contributed to the increased energy loss *via* urinary glucose excretion and mild osmotic diuresis (9, 19). Treatment with SGLT2 inhibitors could also alter body composition through energy loss and osmotic drain, which were associated with fat mass (19). Cefalu and colleagues (25) illustrated that the weight loss observed with canagliflozin in T2DM was mainly due to a reduction in fat mass, with a slightly greater loss of visceral *versus* subcutaneous fat. The reduction in the leptin-adiponectin ratio with remogliflozin has been reported to improve the metabolic health of adults with overweight or obesity but not diabetes, proposing this as an additional mechanism of body weight reduction with SGLT2 inhibitors (19, 26). Furthermore, inhibition of SGLT2 triggered glycogen depletion signals in the liver and activated the liver-brain-adipose axis, resulting in PKA activation in adipocytes, thereby inducing fat reduction and weight loss (27). Further study is required to confirm the potential mechanisms.

A study by Sarich and colleagues (28) showed that canagliflozin increased 24 h urinary glucose excretion in a dose-dependent manner and reduced body weight but was not associated with meaningful changes in plasma glucose or insulin levels in adults with obesity but not diabetes. The adverse events were transient and mild, with no reported hypoglycaemia. Lundkvist and colleagues (29, 30) reported a study of adults with obesity but not diabetes who received dapagliflozin 10 mg once daily and exenatide 2 mg once weekly, acquiring a mean weight loss of −4.5 kg after 24 weeks and −5.7 kg after an additional open-label 28 weeks, and the weight loss was largely due to the reduction in subcutaneous and visceral abdominal adipose tissue. This treatment also had a greater effect on glycaemic control, prediabetes prevalence and SBP. This suggests a potential role for the prevention of T2D and cardiovascular disease in this population.

Although some reviews or meta-analyses have been published before (31–33), this is the first study focusing on the efficacy and safety of SGLT2 inhibitors in adults with overweight or obesity but not diabetes. The retrieved RCTs had mild-to-moderate risks of all biases, and the heterogeneity between each included study was not significant. However, clinical heterogeneity may also exist, including the use of varying types and dosages of SGLT-2 inhibitors among the studies or other baseline and clinical characteristics of the individuals recruited. For example, the majority of the study population was women. Therefore, TSA was used to test the reliability of our study, controlling the potential risk.

There are still certain limitations of this analysis. The major limitation is that we only included six studies with small sample sizes and short follow-up periods, the sample sizes of the included studies ranged from 18 to 376, and the follow-up periods ranged from 2 to 26 weeks, potentially leading to unstable estimates of treatment effects. Additionally, the discontinuation rate was high due to some safety issues. Thirdly, This meta-analysis showed SGLT2 inhibitors can reduce average 1.42kg body weight in adults with overweight or obesity but not diabetes, but the clinical and prognostic benefit of weight change were limited base on the previous studies (34–36). Thus, larger sample sizes with longer durations of observation are needed to clarify the long-term benefits and risks of SGLT2 inhibitors in the treatment of adults with overweight or obesity but not diabetes. Moreover, we were unable to analyse whether the following factors would result in changes in outcomes. For example, the energy intake compensation that occurs in individuals without type 2 diabetes treated with SGLT2 inhibitors over a longer duration have yet to be considered. Only monotherapy and placebo-controlled RCTs were included in this study, and the differences among individual SGLT2 inhibitors cannot be compared due to the lack of head-to-head studies. All the included studies received industry funding, which may produce bias in the results.

In conclusion, SGLT2 inhibitors reduced body weight with statistical significance in adults with overweight or obesity but not diabetes. Given the limited weight reduction and potential harms, selected people may consider SGLT2 inhibitors as an alternative treatment for weight loss in addition to lifestyle intervention when they are at low risk of genital infection and urinary infection. Real world surveillance of the use of SGLT2 inhibitors in people with overweight and obesity but not diabetes is warranted for further information of their effectiveness and safety.

## Data Availability Statement

The original contributions presented in the study are included in the article/**Supplementary Material**. Further inquiries can be directed to the corresponding author.

## Author Contributions

Design: NS and SL. Conduct/data collection: HZ, ML, and NS. Analysis: HZ, ML, and SZ. Writing manuscript: HZ, ML, SL, QS, YZ, SZ, and NS. All authors contributed to the article and approved the submitted version.

## Funding

This study did not receive any grants or funds. Na Su was supported by grants from Health Commission Program (grant number 2020-111) and 1.3.5 Project for Disciplines of Excellence, West China Hospital, Sichuan University (grant number 2018HXFH048).

## Conflict of Interest

The authors declare that the research was conducted in the absence of any commercial or financial relationships that could be construed as a potential conflict of interest.

## Publisher’s Note

All claims expressed in this article are solely those of the authors and do not necessarily represent those of their affiliated organizations, or those of the publisher, the editors and the reviewers. Any product that may be evaluated in this article, or claim that may be made by its manufacturer, is not guaranteed or endorsed by the publisher.

## Supplementary Material

Supplementary Figure 1 Risk of bias graph of all included studies (n=6).

Supplementary Figure 2 Risk of bias summary of all included studies (n=6).

Supplementary Figure 3 Trial sequential analysis of body weight change. RIS, Required information size.

Supplementary Figure 4 Trial sequential analysis of BMI change. RIS, Required information size; BMI, Body mass index.

Supplementary Information 1 Medline Searching strategy.

Supplementary Information 2 Rational of excluding studies.

Supplementary Table 1 PRISMA Checklist for the Meta-analysis.
