# Supplementary figures and images for: Sodium-Glucose Co-Transporter-2 Inhibitors in Non-Diabetic Adults With Overweight or Obesity: A Systematic Review and Meta-Analysis
Source: Front Endocrinol (Lausanne). 2021 Aug 16;12:706914. doi: 10.3389/fendo.2021.706914 (PMC8415407; doi:10.3389/fendo.2021.706914)

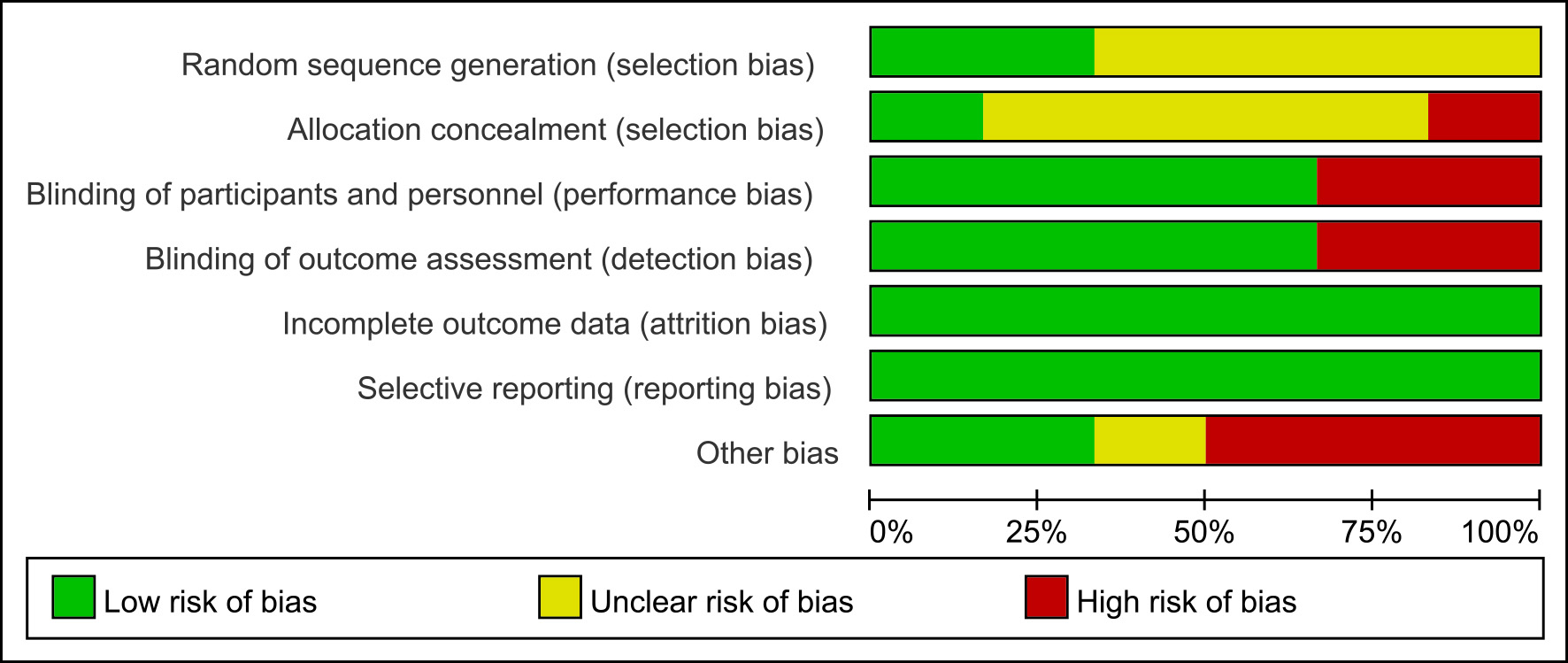

Supplement: Supplementary Figure 1 — Risk of bias graph of all included studies (n=6). [file Image_1.jpeg]

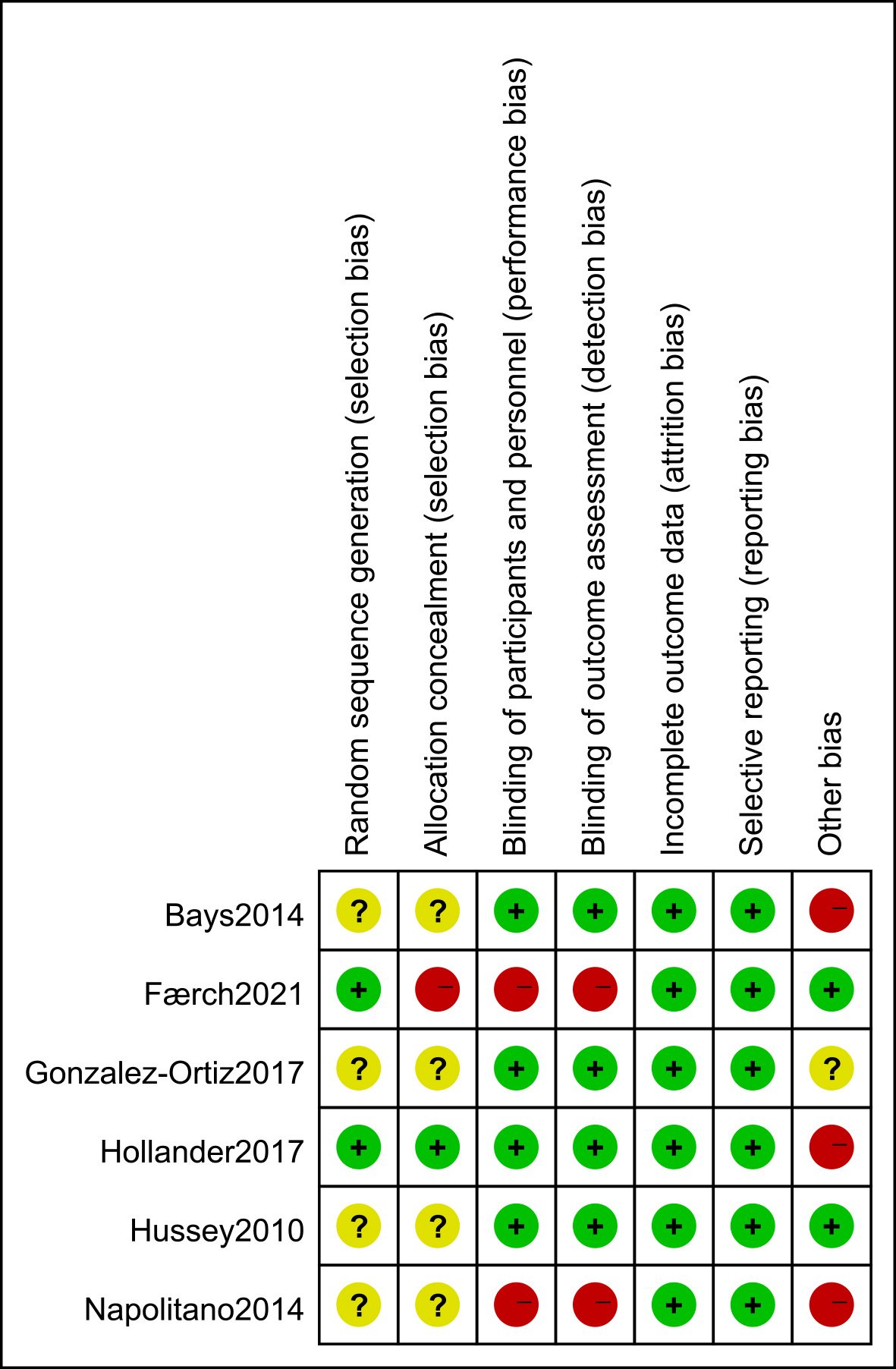

Supplement: Supplementary Figure 2 — Risk of bias summary of all included studies (n=6). [file Image_2.jpeg]

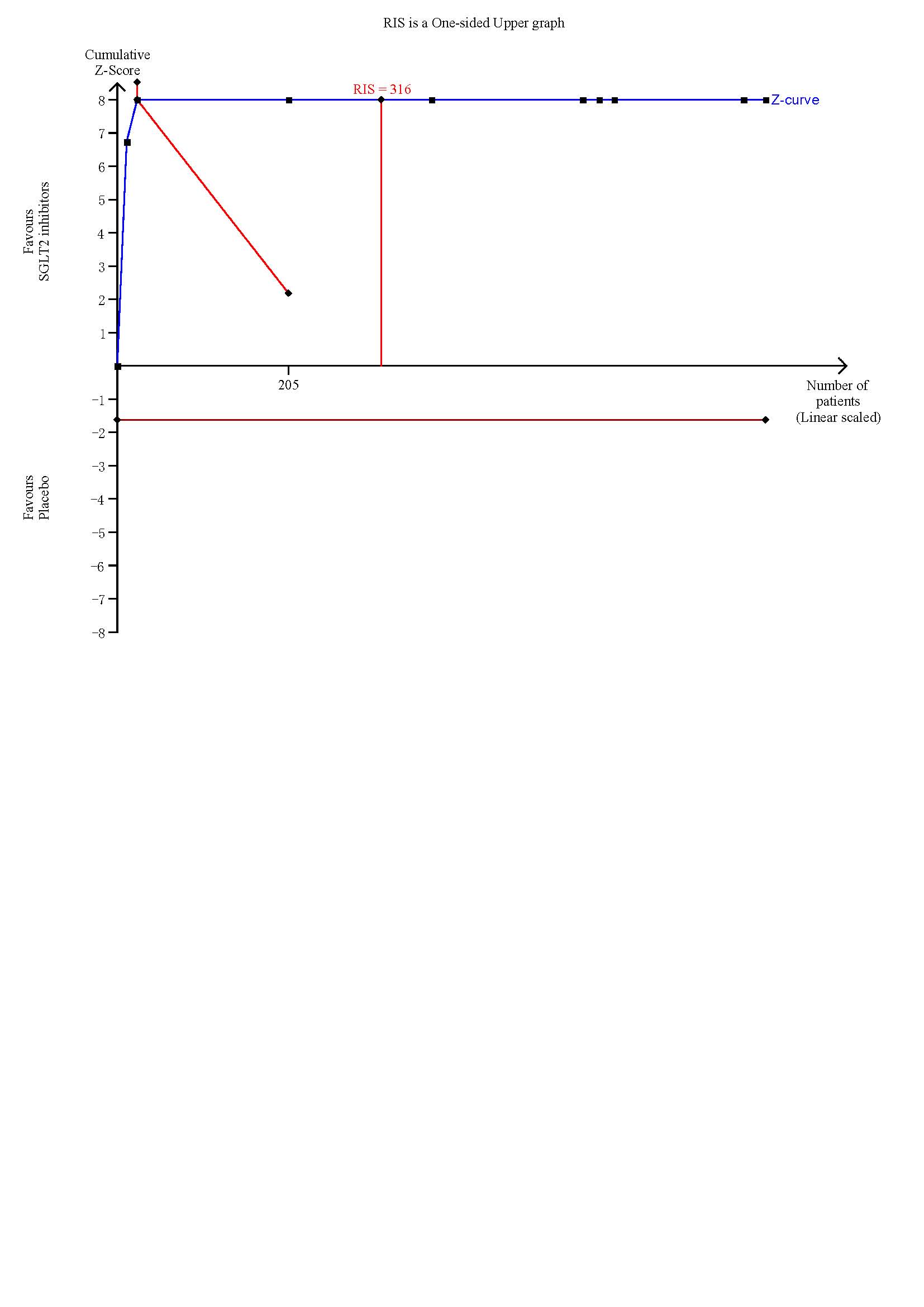

Supplement: Supplementary Figure 3 — Trial sequential analysis of body weight change. RIS, Required information size. [file Image_3.jpeg]

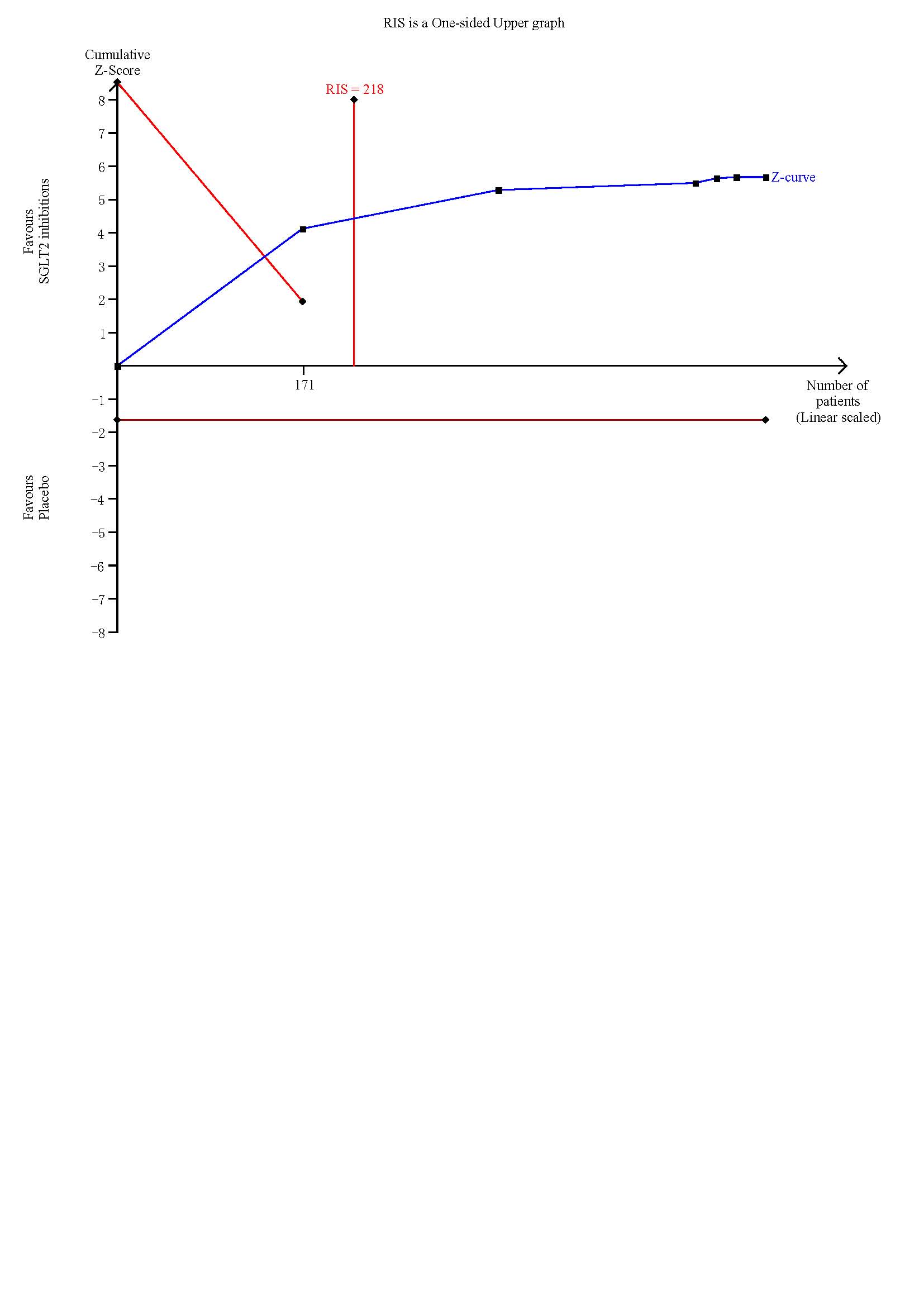

Supplement: Supplementary Figure 4 — Trial sequential analysis of BMI change. RIS, Required information size; BMI, Body mass index. [file Image_4.jpeg]
