# Supplementary material for: Sodium-Glucose Co-Transporter-2 Inhibitors in Non-Diabetic Adults With Overweight or Obesity: A Systematic Review and Meta-Analysis
Source: Front Endocrinol (Lausanne). 2021 Aug 16;12:706914. doi: 10.3389/fendo.2021.706914 (PMC8415407; doi:10.3389/fendo.2021.706914)
Supplement: Supplementary Information 1 — Medline Searching strategy. [file DataSheet_1.pdf]

Supplementary information 1. MEDLINE Searching strategy

1. exp Sodium-Glucose Transporter 2/
2 Sodium glucose cotransporter 2 inhibitor.tw.
3 canagliflozin.tw.
4 dapagliflozin.tw.
5 empagliflozin.tw.
6 ipragliflozin.tw.
7 tofogliflozin.tw.
8 luseogliflozin.tw.
9 sergliflozin.tw.
10 remogliflozin.tw.
11 ertugliflozin.tw.
12 sotagliflozin.tw.
13 exp Overweight/
14 exp Obesity/
15 Overweight.tw.
16 Obesity.tw.
17 obese.tw.
18 1 or 2 or 3 or 4 or 5 or 6 or 7 or 8 or 9 or 10 or 11or 12
19 13 or 14 or 15 or 16 or 17
20 18 and 19
