# Supplementary material for: Sodium-Glucose Co-Transporter-2 Inhibitors in Non-Diabetic Adults With Overweight or Obesity: A Systematic Review and Meta-Analysis
Source: Front Endocrinol (Lausanne). 2021 Aug 16;12:706914. doi: 10.3389/fendo.2021.706914 (PMC8415407; doi:10.3389/fendo.2021.706914)
Supplement: Supplementary Information 2 — Rational of excluding studies. [file DataSheet_2.pdf]

Supplementary information 2. Rationale of excluding studies

Study	Reason for exclusion
Neeland2020	Did not measure any outcome of interest
Javed2020	Did not measure any outcome of interest
Pereira2017	Combine therapy (Dapagliflozin 10 mg qd +exenatide 2 mg qd in experiment group)
Kato 2017	Overweight patients with type 2 diabetes: a randomized, crossover, controlled clinical trial.
Lundkvist 2017	Combine therapy (Dapagliflozin 10 mg qd +exenatide 2 mg qd in experiment group)
Lundkvist 2017	Combine therapy (Dapagliflozin 10 mg qd +exenatide 2 mg qd in experiment group)
Rosenstock 2014	obese inadequately controlled type 2 diabetes
Sarich 2010	Published as abstract only.
Deol 2017	diabesity, the real world experience, Combination therapy with GLP-1 analogues ,with no control
Faerch 2017	Protocol, prediabetes
NCT02811484	Withdrawn, Patients With Type 2 Diabetes
NCT02360774	not completed
NCT02338193	not completed
NCT02243202	Overlapping population
NCT01018823	Completed with no result
NCT00650806	Overlapping population

Exclude studies

1. Neeland IJ, Albuquerque Rocha ND, Hughes C, Ayers CR, Malloy CR, Jin ES.Effects of Empagliflozin Treatment on Glycerol-Derived Hepatic Gluconeogenesis in Adults with Obesity: A Randomized Clinical Trial.Randomized Controlled Trial Obesity (Silver Spring). 2020;28:1254-1262.
2. Javed Z, Papageorgiou M, Madden LA, Rigby AS, Kilpatrick ES, Atkin SL,Sathyapalan L.The effects of empagliflozin vs metformin on endothelial microparticles in overweight/obese women with polycystic ovary syndrome.Endocr Connect. 2020;9(6):563-569.

3. Pereira MJ, Lundkvist P, Kamble PG, Lau J, Martins JG, Sjöström CD, Schnecke V, Walentinsson A, Johnsson E, Eriksson JW.A Randomized Controlled Trial of Dapagliflozin Plus Once-Weekly Exenatide Versus Placebo in Individuals with Obesity and Without Diabetes: Metabolic Effects and Markers Associated with Bodyweight Loss.Diabetes Ther. 2018;9:1511-1532.
4. Kato K, Suzuki K, Aoki C, Sagara M, Niitani T, Wakamatsu S, et al. The effects of intermittent use of the SGLT-2 inhibitor, dapagliflozin, in overweight patients with type 2 diabetes in Japan: a randomized, crossover, controlled clinical trial. Expert Opinion on Pharmacotherapy. 2017;18:743-51.
5. Lundkvist P, Sjostrom CD, Amini S, Pereira MJ, Johnsson E, Eriksson JW. Dapagliflozin once-daily and exenatide once-weekly dual therapy: A 24-week randomized, placebo-controlled, phase II study examining effects on body weight and prediabetes in obese adults without diabetes. Diabetes, Obesity and Metabolism. 2017;19:49-60.
6. Lundkvist P, Pereira MJ, Katsogiannos P, Sjostrom CD, Johnsson E, Eriksson JW. Dapagliflozin once daily plus exenatide once weekly in obese adults without diabetes: Sustained reductions in body weight, glycaemia and blood pressure over 1 year. Diabetes, Obesity & Metabolism. 2017;19:1276-88.
7. Rosenstock J, Jelaska A, Frappin G, Salsali A, Kim G, Woerle HJ, et al. Improved glucose control with weight loss, lower insulin doses, and no increased hypoglycemia with empagliflozin added to titrated multiple daily injections of insulin in obese inadequately controlled type 2 diabetes. Diabetes Care. 2014;37:1815-23.
8. Sha S, Devineni D, Ghosh A, Polidori D, Chien S, Wexler D, Shalayda K, Demarest K, Rothenberg P. Canagliflozin, a novel inhibitor of sodium glucose co-transporter 2, dose dependently reduces calculated renal threshold for glucose excretion and increases urinary glucose excretion in healthy subjects. Diabetes, Obesity & Metabolism. 2011; 13: 669-72.
9. Deol H, Lekkakou L, Viswanath AK, Pappachan JM. Combination therapy with GLP-1 analogues and SGLT-2 inhibitors in the management of diabesity: the real world experience. Endocrine. 2017;55:173-8.
10. Faerch K, Amadid H, Nielsen LB, Ried-Larsen M, Karstoft K, Persson F, et al. Protocol for a randomised controlled trial of the effect of dapagliflozin, metformin and exercise on glycaemic variability, body composition and cardiovascular risk in prediabetes (the PRE-D

Trial. BMJ Open. 2017,7.

11. Weill Medical College of Cornell University. Efficacy of Exenatide-LAR and Dapagliflozin in Overweight/Obese, Insulin Treated Patients With Type 2 Diabetes. https://www.clinicaltrials.gov. NCT02811484.
12. Beth Israel Deaconess Medical Center, Janssen Scientific Affairs, LLC. Mechanisms of Weight Loss With SGLT2 Inhibition. https://www.clinicaltrials.gov. NCT02360774.
13. AstraZeneca. Dapagliflozin and Metformin,Alone and in Combination, in Overweight/Obese Prior GDM Women. https://www.clinicaltrials.gov. NCT02338193.
14. Janssen Research & Development, LLC. 2Effects of Co-administration of Canagliflozin 300 mg and Phentermine 15 mg With Placebo in the Treatment of Non-Diabetic Overweight and Obese Participants. https://www.clinicaltrials.gov. NCT02243202.
15. Merck Sharp & Dohme Corp, Pfizer. 3A Multiple Dose Study Of Ertugliflozin(PF-04971729, MK-8835) In Otherwise Healthy Overweight And Obese Volunteers (MK-8835-037). https://www.clinicaltrials.gov. NCT01018823.
16. Johnson & Johnson Pharmaceutical Research & Development, L.L.C. A Study of the Safety and Effectiveness of Canagliflozin (JNJ-28431754) in Promoting Weight Loss in Overweight and Obese Patients Who do Not Have Diabetes. https://www.clinicaltrials.gov. NCT00650806.
